# Protective Role of Decorin in Primary Hepatocellular Carcinoma

**DOI:** 10.3389/fonc.2020.00645

**Published:** 2020-05-12

**Authors:** Andrea Reszegi, Zsolt Horváth, Hajnalka Fehér, Barnabás Wichmann, Péter Tátrai, Ilona Kovalszky, Kornélia Baghy

**Affiliations:** ^1^1st Department of Pathology and Experimental Cancer Research, Semmelweis University, Budapest, Hungary; ^2^2nd Department of Internal Medicine, Semmelweis University, Budapest, Hungary; ^3^Solvo Biotechnology, Budapest, Hungary

**Keywords:** decorin (DCN), hepatocellular carcinoma, proteoglycan (PG), hepatocarcinogenesis, extracellular matrix (ECM)

## Abstract

Hepatocellular carcinoma (HCC) represents one of the most frequent type of primary liver cancers. Decorin, a small leucine-rich proteoglycan of the extracellular matrix, represents a powerful tumor cell growth and migration inhibitor by hindering receptor tyrosine kinases and inducing p21^WAF1/CIP1^. In this study, first we tested decorin expression in HCCs utilizing *in silico* data, as well as formalin fixed paraffin embedded tissue samples of HCC in a tissue microarray (TMA). *In silico* data revealed that DCN/SMA mRNA ratio is decreased in HCC compared to normal tissues and follows the staging of the disease. Among TMA samples, 52% of HCCs were decorin negative, 33% exhibited low, and 15% high decorin levels corroborating *in silico* results. In addition, applying conditioned media of hepatoma cells inhibited decorin expression in LX2 stellate cells *in vitro*. These results raise the possibility that decorin acts as a tumor suppressor in liver cancer and that is why its expression decreased in HCCs. To further test the protective role of decorin, the proteoglycan was overexpressed in a mouse model of hepatocarcinogenesis evoked by thioacetamide (TA). After transfection, the excessive proteoglycan amount was mainly detected in hepatocytes around the central veins. Upon TA-induced hepatocarcinogenesis, the highest tumor count was observed in mice with no decorin production. Decorin gene delivery reduced tumor formation, in parallel with decreased pEGFR, increased pIGF1R levels, and with concomitant induction of pAkt (T308) and phopho-p53, suggesting a novel mechanism of action. Our results suggest the idea that decorin can be utilized as an anti-cancer agent.

## Introduction

Hepatocellular carcinoma (HCC) is one of the most common primary liver malignancy and it is the fourth cancer-related death cause in the world. Incidence of HCC is higher among males than females, it occurs mainly in Northern and Western Africa (Egypt, the Gambia, Guinea) Eastern- and South-Eastern Asia (Mongolia, Cambodia, and Vietnam), Melanesia, and Micronesia/Polynesia (1). Hepatitis B (HBV) or hepatitis C virus (HCV) infection, aflatoxin, smoking, type 2 diabetes, and alcohol abuse represent the main risk factors for the development of HCC (1, 2). In HCC, chronic inflammation usually precedes malignant transformation progressing from fibrosis to cirrhosis and tumor formation (2–4). However, a small fraction of cases occurs without cirrhosis. HCC can be treated curatively with surgical resection or liver transplantation if diagnosed at an early stage (2–4). Surgery can only be performed in about 15% of patients and they generally have a poor prognosis with median survival times of <1 year (2, 3).

The extracellular matrix (ECM) is a highly dynamic structure that is present in all tissues and continuously undergoes controlled remodeling (5). ECM macromolecules exhibit important functional roles in the control of several cellular events such as adhesion, migration, proliferation, differentiation, and survival (5–7). The matrix is well known for its ability to provide structural and biochemical support for organs and tissues. The ECM is composed of collagens, elastin, proteoglycans (PGs), and non-collagenous glycoproteins (5, 6).

Matrix remodeling plays an important role in the development of HCC. This process involves quantitative and qualitative changes in the ECM (6). Tumor cells can manipulate their microenvironment to enhance their own survival, thereby creating a positive tumorigenic feedback loop (8). Accordingly, during the last decades extensive research activities focused on the better understanding of the cancer cell and stroma interactions.

Decorin is a member of the ECM small leucine-rich proteoglycan (SLRP) gene family (9–11) containing a single chondroitin sulfate or dermatan sulfate chain and is expressed mainly by fibroblast and myofibroblasts (12–14). In healthy liver, a small amount of decorin is deposited around the central veins and in the portal tracts. However, during fibrogenesis together with other matrix proteins the amount of decorin significantly increases in the connective tissue septa (14–16). Decorin has been described to be involved in many biological and physiological processes including growth regulation (17–20), cell differentiation (19), collagen fibrillogenesis (21–24), muscular development (25), wound healing (26), stem cell biology (27), kidney and liver fibrosis (28, 29), angiogenesis (30), regulation of inflammation and autophagy (31).

Decorin represents a powerful tumor cell growth and migration inhibitor by interaction with matrix constituents and regulating several signaling pathways (19). The first growth factor discovered as a decorin interacting partner was the transforming growth factor-β (TGF-β). Binding of TGF-β by the proteoglycan attenuates proliferation of tumor cell lines dependent on the growth factor (18, 32). Previous studies have shown that decorin is an endogenous, soluble pan-receptor tyrosine kinase (RTK) inhibitor, known to interact with variety of cell surface receptors including epidermal growth factor receptor (EGFR/ErbB1) (9) as well as another members of the ErbB RTK family (33–35). Moreover, decorin negatively regulates insulin-like growth factor receptor I (IGF-IR) (36–39), the hepatocyte growth factor receptor Met (40), vascular endothelial growth factor receptor 2 (VEGFR-2) (41) and platelet-derived growth factor receptor (PDGFR) (14).

To better understand the role of decorin in HCC, the aim of this study was to examine the expression of decorin in liver tumor using *in silico* approaches as well as FFPE tissue microarray (TMA) samples of HCC with or without cirrhosis. Our previous studies (14, 42) showed that the lack of decorin favors primary hepatocarcinogenesis resulting in higher tumor incidence. In addition, decorin expression is decreased in HCC. Thus, to confirm the protective role of decorin in the other way around, we designed a model system to investigate the effects of overexpressed decorin in mouse model of hepatocarcinogenesis evoked by thioacetamide (TA).

## Materials and Methods

### Data Acquisition and Preprocessing

The gene expression datasets for HCC and non-tumorous liver samples were collected from the public microarray repository ArrayExpress database (43), provided by the European Bioinformatics Institute (Saffron Walden, UK). Our datasets with accession E-MTAB-950 (https://www.ebi.ac.uk/arrayexpress/experiments/E-MTAB-950/) includes 36 normal, 112 tumors, and 10 pair of tumors–non-tumorous adjacent tissues (NATs). Most of the HCC patients have the underlying etiology of Hepatitis C Virus and Hepatitis B virus infection. All the raw data were processed using R programming language due to its detailed clinicopathological data.

### Tissue Microarray (TMA)

Tissue blocks were collected from the Biopsy archive of the 1st Department of Pathology and Experimental Cancer Research, Semmelweis University. The FFPE tissue samples were used with the approval of Semmelweis University Regional and Institutional Committee of Science and Research Ethics (TUKEB permit number: 95/1999). Representative normal and tumorous areas were selected by two independent pathologists for TMA construction. We utilized FFPE tissue samples of HCC with and without cirrhosis. Biopsy samples of 29 HCCs (20 cirrhotic, 9 non-cirrhotic) and 9 control livers (hemangioma) were selected for TMA assembly. A detailed list of biopsy samples is provided in Table S1. From each HCC, one core from the tumor and one from the non-tumorous adjacent tissue (NAT) was selected. TMA block was sectioned, and slides were immunostained for decorin and smooth muscle actin (SMA) (Table S2). Staining intensities were analyzed by Pannoramic Viewer software using a 12-score system and evaluated by two independent pathologists visual scoring. Every sample was given a score according to the intensity of the decorin staining (no staining = 0, low decorin staining = 1–6, and high decorin staining = 7–12). The final label is determined by averaging two pathologists' scores. HCC samples were divided into decorin negative, low and high decorin expressing categories. To compensate for the variation of fibroblast content, decorin expressions were normalized to SMA content.

### Immunostaining

Immunohistochemistry was performed on FFPE sections, and fluorescent staining was made on methanol-acetone-fixed liver tissues according to standard protocols (42). Antibodies specifications and dilutions are listed in Table S2.

### Real Time q-PCR

For RT-qPCR, total RNA was isolated from macro-dissected FFPE liver tissue samples and treated LX2 cells. After homogenization, total RNA was purified using the PureLink FFPE Total RNS isolation kit (Life Technologies, Carlsbad CA, USA) for FFPE samples, and RNEasy Mini kit (Qiagen, Hilden, Germany) for cell samples according to the protocols provided by the manufacturers. The integrity of the total RNA was analyzed on the Experion Automated Electrophoresis Station (Bio-Rad Laboratories GmbH, Münich, Germany).

Total RNA reverse transcription and RT-qPCR from samples were done as detailed previously (42). RT-qPCR was accomplished by using TaqMan Gene Expression Assays for human: decorin (DCN, Assay ID: Hs00370383_m1, Life Technologies) and human smooth muscle actin (ACTA2, Assay ID: Hs.PT.56a21389192) according to the manufacturer's protocol. Human glyceraldehyde 3-phosphate dehydrogenase (GAPDH) (GAPDH, Assay ID: Hs.PT.39a22214836, Integrated DNA Technologies) and 18S RNA (Part No.:4319413E) were used as endogenous controls. All samples were run in duplicates. Results were obtained as threshold cycle values. Expression levels were determined by using the 2^−ΔΔCT^ method.

### Tissue Culture and Reagents

LX2 human hepatic stellate cell line was provided by Dr. Scott Friedman, HepG2, and Hep3B cell lines were obtained from the American Type Culture Collection (Manassas, VA), HuH7 and HLE were acquired from the Japanese Collection of Research Bioresources Cell Bank (Osaka, JP). Cells were cultured in Dulbecco's modified Eagle's medium (DMEM-1000) (Sigma Aldrich, St. Louise, MO, USA) with 1,000 mg/l (5.5 mmol/l) glucose concentration, supplemented with 10% [v/v] fetal bovine serum albumin (FBS, Sigma Aldrich), and 1% [v/v] Penicillin/Streptomycin (Sigma Aldrich) in an atmosphere containing 5% CO_2_ at 37°C.

To obtain cell conditioned medium (CM), hepatoma cell lines (HepG2, Hep3B, Huh7, HLE) were cultured as described above until 80% confluence and then the medium was changed with fresh DMEM. CM was harvested and stored after 16 h.

LX2 cells were grown to 80% confluency in 6-well-plate. At that time cells were exposed to hepatoma-CM for 24 h, then starved overnight in FBS-free DMEM. LX2 cells with only FBS-free medium served as control. After treatment, both cells and supernatants were saved for protein and mRNA studies. Experiments were repeated three independent times.

### Phospho-Kinase Array, Western Blot, and Dot Blot

For phospho-kinase array, western blot, and dot blot analyses, frozen liver samples and cells were extracted in lysis buffer containing 20 mM TRIS pH = 7.5, 2 mM EDTA, 150 mM NaCl, 1% Triton X-100, 0.5% Protease Inhibitor Cocktail (Sigma, St. Luis, MO, USA), 2 mM Na_3_VO_4_, 10 mM NaF. Western blot and dot blot analyses were prepared as previously indicated (42, 44). For Western blotting, 20 μg of proteins were loaded per lane and for dot blot analysis 200 μl cell culture media was applied on PVDF membrane. Antibodies specifications and dilutions are listed in Table S2. Western and dot blot analyzes were performed three independent times.

The activities of phospho-kinases were checked by using the Proteome Profiler Phospho-Kinase Array Kit (R&D Systems, Minneapolis, USA) according to manufacturer's user guide. In brief, pooled samples of four livers from the same experimental group were homogenized in lysis buffer (described above) and adjusted to 1,000 μg of protein per 2,000 μl lysate. Signals of the Western blot, dot blot, and array membranes were detected by SuperSignal West Pico Chemiluminescent Substrate Kit (Thermo Fisher Scientific Inc., Waltham, USA), and visualized on iBright FL1500 Imaging System (Thermo Fisher Scientific).

### DNA Plasmid

We used pLIVE expression vector (Liver *in vivo* Expression; Mirus Bio, Madison, WI, USA) to achieve high level and prolonged transgene expression in the mouse liver. The vector is driven by a liver-specific chimeric promoter composed of the mouse α-fetoprotein enhancer II and the minimal mouse albumin promoter. In addition, pLIVE-SEAP (secreted alkaline phosphatase) vector was created for use as positive controls. Expression of the SEAP protein from pLIVE-SEAP can be easily monitored using a quantitative chemiluminescence assay of mouse serum.

Full-length cDNA of human decorin (DCN) gene inserted into the pGEM-1 expression vector was subcloned in the pLIVE vector using BamHI and XhoI restriction sites. The insertion was confirmed by DNA sequencing (Semmelweis University). Plasmid DNA was amplified in Escherichia coli DH5α cells and isolated by alkaline lysis and subsequently purified by an anion exchange resin column according to the manufacturer's instructions (Qiagen, Valencia, CA). The quality and quantity of the plasmid DNA was analyzed by restriction endonuclease digestion, agarose gel electrophoresis and absorbance at 260/280 nm by ND-1000 spectrophotometer (NanoDrop Technologies, Wilmington, DE, USA).

### Hydrodynamic Gene Delivery

All animal study protocols were conducted according to the ethical standards of the Animal Health Care and Control Institute, Csongrád County, Hungary. All animal experiments were approved by the following ethical license: XVI/ 03047-2/2008.

Thirty-six years two-month-old, 18–25 g male wild-type C57BL/6 mice were used for our experiments. Plasmid DNA [pLIVE-SEAP together with pLIVE-DCN, or pLIVE-SEAP with pLIVE-0 (control)] was injected by hydrodynamic technique according to the manufacturer's instructions (Mirus Bio LLC). In brief, 15 μg of high quality/purity plasmid DNA was prepared in 2 ml of pharmaceutical grade saline solution at room temperature. Mice were anesthetized, and the lateral tail vein was accessed using a 27-gauge needle (according Mirus Bio LLC). Administration of the solution was performed in 4–7 s, at a constant rate, without extravasation. Each group was represented by 18 animals.

### Induction of Experimental Hepatocarcinogenesis by Thioacetamide Treatment

Induction of liver cancer was performed as previously described (42). In brief, we utilized a total of 30 years 2-month-old male mice all in a C57Bl/6 background. Mice were subjected to TA treatment for 10 months. Age-matched untreated animals with identical genetic background served as controls. Blood samples were collected at the half time and at the end of the treatment. At termination, half of the liver samples were fixed in formalin and embedded in paraffin for histological analysis and the other half was frozen for further experiments.

### SEAP Reporter Gene Assay

SEAP activity from half-time treated mouse serum was measured using the Phospha-Light™ SEAP Reporter Gene Assay kit (Thermo Fisher Scientific), according to the manufacturer's datasheet.

Chemiluminescent plates were visualized by Kodak Image Station 4000MM Digital Imaging System. The density of the dots was quantified using the free ImageJ (Version 1.50b, NIH, USA) software. Each assay was performed in duplicate, and the mean values were used for statistical analysis.

### Enzyme-linked Immunosorbent Assay (ELISA)

The human decorin levels from half-time treated mice serum were quantified by sandwich enzyme-linked immunosorbent assay, using the Human Decorin ELISA Kit from Sigma-Aldrich (Cat.No. #RAB0140 Sigma, St. Luis, MO USA), according to the manufacturer's instructions. Samples were evaluated from 10 mice per group. Each sample was performed in duplicate and the mean values were used for statistical analysis. ELISA plates were read at 570 nm with Labsystem Multiscan MS 352 (Labsystems, Finland) plate reader.

### Statistical Analysis

All statistical analyses were performed by Graphpad Prism 4.03 software (Graphpad Software Inc., La Jolla, CA, USA). Data evaluation was performed using D'Agostino and Pearson's omnibus normality test and non-parametric tests (Mann–Whitney) or Students' *t*-tests depending on the distribution of the data. The difference between control and DCN treated groups in tumor prevalence was tested for significance by χ^2^-test. *P* < 0.05 level was declared statistically significant.

## Results

### Downregulation of Decorin in Human Hepatocellular Carcinoma *in silico* Experiments

**Figure 1:**
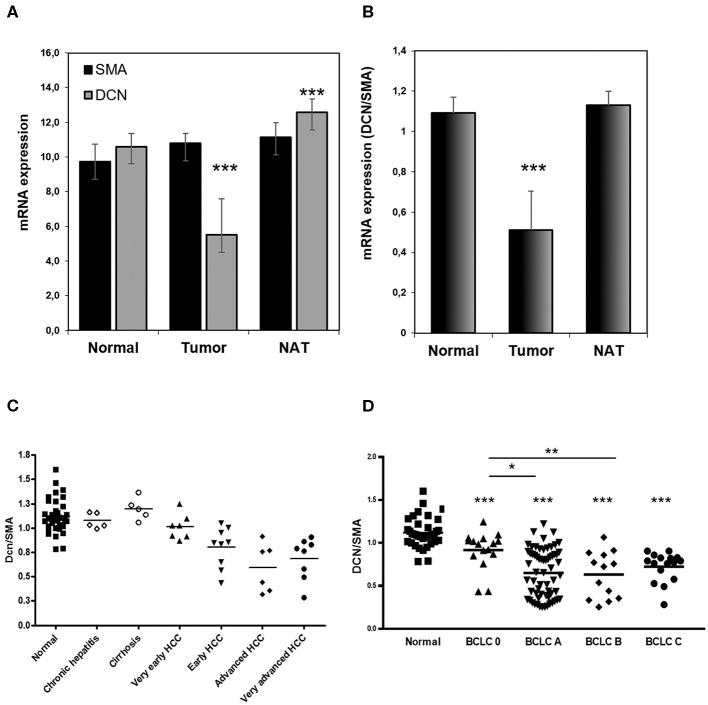
Distribution of human decorin in hepatocellular carcinoma. DCN/SMA content separates normal and cancerous samples. Decorin expression is extremely low in tumor samples compared to normal and NAT sections **(A,B)**. DCN expression seems to follow the classification of BCLC staging **(D)** and aggressiveness of HCC **(C)** [(*n*_normal_ = 36, *n*_HCC_ = 112)]. DCN, decorin; NAT, non-tumorous adjacent tissue; SMA, smooth muscle actin. All data are presented as mean of normalized ± SD. ***p < 0.001; **p < 0.01; *p < 0.05.

Analysis of HCC cases revealed that tumor samples had significantly decreased decorin mRNA expression compared to normal liver (*p* < 0.001) and displayed moderate increases in NATs (*p* < 0.001; Figure 1A). When normalized to SMA content, decorin expression was significantly reduced in tumor samples compared to normal tissue and NAT sections (*p* < 0.001) (Figures 1A,B). However, no difference in the normalized proteoglycan level of NAT and normal tissue was revealed (Figure 1B). According to the *in silico* analysis, DCN/SMA content distinguishes between normal and cancerous samples, and is even characteristic for very early stage HCC (Figure 1C). DCN/SMA ratio gradually decreases from very early to advanced HCC, while it is overexpressed in cirrhosis (Figure 1C).

In addition, decorin expression seems to follow the BCLC (Barcelona Clinic Liver Cancer) staging classification as significantly decreased decorin level was observed in every BCLC stage compared to normal liver (*p* < 0.001), and its level gradually decrease from BCLC 0 to BCLC B (*p* < 0.05 and 0.01; Figure 1D). All data of the TMA study are presented in Table S3.

### Inhibited Decorin Production of Fibroblasts at Protein Level in HCC

Next, we aimed to detect changes in decorin expression at protein level. To this end, we utilized FFPE HCC tissue samples with or without cirrhosis. From each HCC sample, one core from the tumor and one from NAT was selected and immunostaining specific for decorin and SMA was performed. Decorin and SMA mRNA levels were determined by RT-qPCR analyzes.

**Figure 2:**
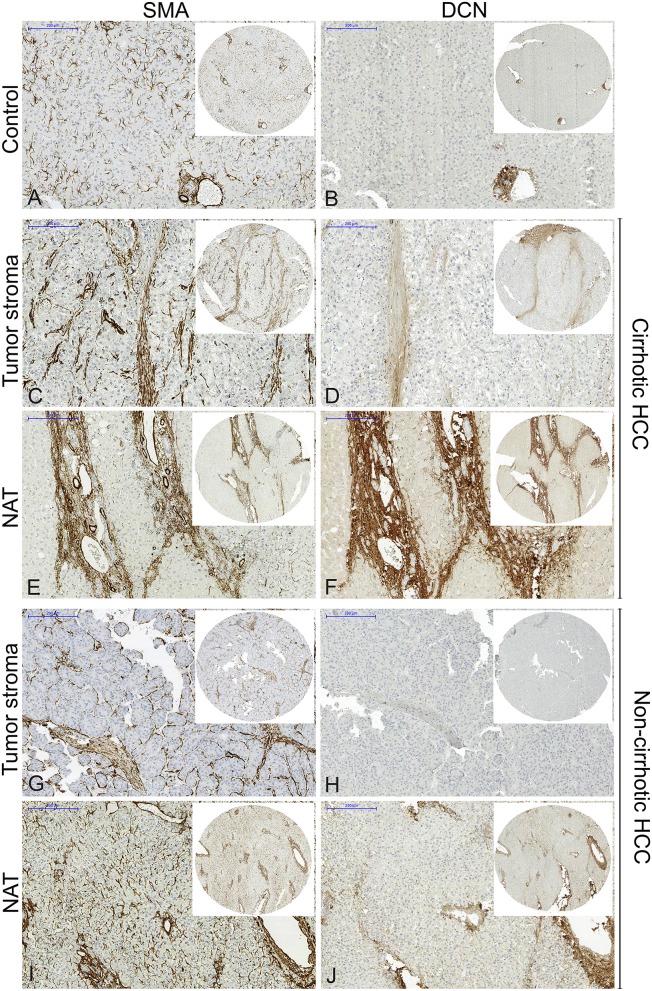
Representative images of decorin and SMA immunostaining of normal liver **(A,B)**, cirrhotic HCC **(C–F)**, and non-cirrhotic HCC **(G–J)**. From each HCC sample, one core from the tumor and one from NAT tissue was selected. Decreased decorin expression was detected in tumor stroma, compared to NAT both at protein and mRNA levels, which may reflect on the aggressiveness of the HCC. NAT, non-tumorous adjacent tissue; SMA, smooth muscle actin. Scale bar 200 μm. *n* = 27.

Immunohistochemical staining of SMA reflects on the number of activated hepatic stellate cells (HSCs), the main source of decorin in the liver. In the normal human liver, SMA is localized in the perisinusoidal area as well as in the vascular walls of the portal tract and the central vein (Figure 2A). In control liver, weak immunopositivity of decorin was detected around the central veins and in the portal tracts (Figure 2B). DAB positivity for SMA in cirrhotic and non-cirrhotic liver samples are strongly and diffusely located in cytoplasm of fibroblasts in connective tissue septa and in the perisinusoidal spaces of residual hepatic parenchyma (Figures 2C,E,G,I).

In the NAT of both cirrhotic and non-cirrhotic HCCs, a high number of α-SMA-positive activated HSCs were detected (Figures 2E,I) with extremely strong decorin expression (Figures 2F,J) along the sinusoids, the portal tracts and around the central veins in the same tissue section. In contrast, a high number of α-SMA-positive activated HSCs were detected in the tumor stroma (Figures 2C,G), but there was hardly any, or negative decorin expression (Figures 2D,H) in the same sample. This observation was detected in both cirrhotic and non-cirrhotic cases.

**Figure 3:**
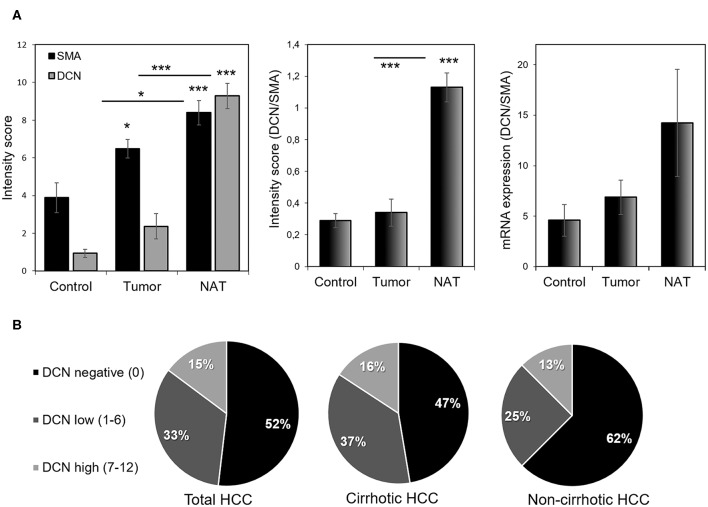
Representative images of the normalized decorin and SMA expression. We normalized decorin expression to SMA content in order to avoid distortion of results by the different number of decorin producing myofibroblasts **(A)**. Note the low decorin expression in tumor despite of the large number of SMA-positive MFs **(A)**. Based on immunoscores, HCCs were categorized as negative, low, and high decorin expressing tumors **(B)**. Most HCCs lack or under-express of decorin **(B)**. NAT, non-tumorous adjacent tissue; SMA, smooth muscle actin. *n* = 27. All data are demonstrated as mean of normalized ± SD. ***p < 0.001; *p < 0.05.

Immunohistochemical results were semi-quantified using a 12-score system and evaluated by visual scoring of two independent pathologists. Significantly increased decorin and SMA levels were observed in NAT samples compared to normal liver (*p* < 0.001 for both decorin and SMA) and tumor stroma (*p* < 0.05 for SMA; Figure 3A). Tumor samples contained significantly less decorin and SMA than that of NAT (*p* < 0.05 for SMA and *p* < 0.001 for decorin; Figure 3A). When normalized to SMA content, decorin expression both at protein and mRNA level was decreased in the tumor samples compared to their paired NAT. At protein level, the difference was statistically significant (*p* < 0.001; Figure 3A). As decorin expression was normalized to SMA level, differences were not caused by changes in the number of myofibroblast cells.

Based on their intensity score, HCC samples were divided into decorin negative, low and high expressing categories. Using this evaluation, 52% of HCCs were decorin negative, 33% showed low, and 15% high decorin expression (Figure 3B). Negativity and low expression were more characteristic for HCCs without cirrhosis (Figure 3B).

### Tumor Cells Inhibit Decorin Production of LX2 Stellate Cells *in vitro*

**Figure 4:**
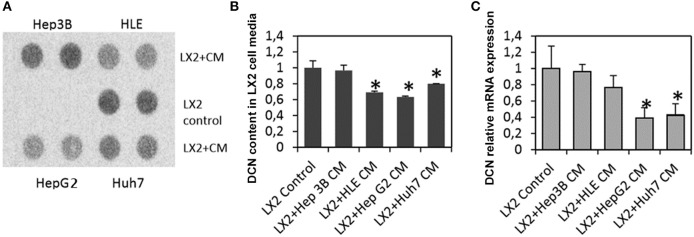
Decorin production of LX2 stellate cells upon exposure to hepatoma (Hep3B, HLE, HepG2, and HuH7) cell medium. Dot blot analysis of decorin content in LX2 cell media **(A)** and its quantification **(B)**. Determination of decorin mRNA levels **(C)**. CM, conditioned medium. Data are presented as mean of normalized ± SD. *p < 0.05.

To test whether tumor cells are capable of directly influence the decorin production of myofibroblasts, LX2 human stellate cells were exposed to conditioned media of different hepatoma cell lines (Hep3B, HLE, HepG2, and HuH7). Significantly less decorin was detected in the media of LX2 cells, when HLE, HepG2, and HuH7 conditioned media was applied (*p* < 0.05; Figures 4A,B). In case of Hep3B cells, the observed effect did not reach statistical significance. These changes appeared at transcriptional level, as decorin mRNA level was significantly reduced in LX2 cells exposed to HepG2, HuH7 conditioned media (*p* < 0.05) (Figure 4C). Decorin mRNA level was also reduced, when Hep3B and HLE conditioned media was applied, but these changes were not statistically significant. These results correlate well with our observations on human HCC tissue samples indicating that the presence of tumor cells reduces the expression of decorin highlighting its tumor suppressor effect in HCC.

### Conformation of Successful Decorin Gene Delivery and Expression in Experimental Liver Cancer

Our previous studies showed that the lack of decorin favors primary hepatocarcinogenesis resulting in higher tumor incidence (14, 42). Based on these findings, we designed a new set of experiments to understand the implication of overexpressed decorin in our TA-induced hepatocarcinogenesis model. For this experiment, human decorin cDNA was cloned into a pLIVE vector, where the expression is driven by a mouse AFP enhancer and albumin promoter. In addition, we applied a control vector coding serum alkaline phosphatase. When injecting together with the human decorin-coding (pLIVE-DCN) or with the empty vector (pLIVE-0), the SEAP detected from blood provides indirect information about the activity of the pLIVE-DCN or pLIVE-0 vectors. Vectors were injected using hydrodynamic gene delivery method.

**Figure 5:**
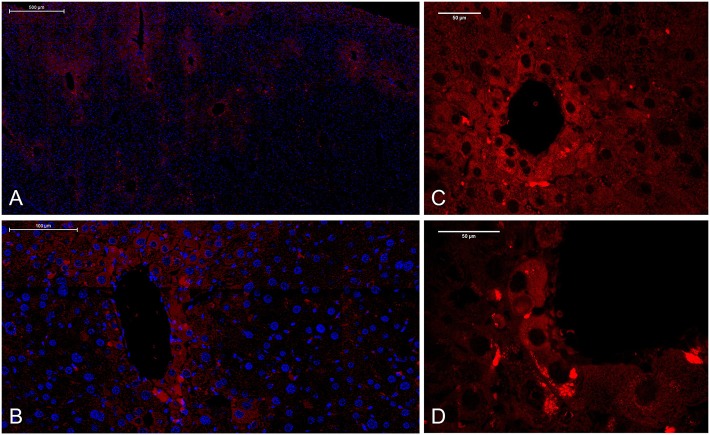
Localization of human recombinant decorin in pLIVE-DCN-transfected liver sections. Fluorescent immunostaining of overexpressed decorin (red) after hydrodynamic gene delivery. Nuclei were counterstained with DAPI (blue). **(A)** Scale bar = 500 μm, **(B)** 100 μm, and **(C,D)** 50 μm.

The artificial decorin expression and localization was visualized by fluorescent immunostaining (Figure 5A). Human decorin was successfully transfected and expressed in the livers. Control livers transfected with the empty vector (pLIVE-0) were completely negative for immunostaining of human decorin. Driven by albumin promoter, the human recombinant decorin produced by the transfected vector was mainly detected in hepatocytes around the central veins (Figures 5A,B) and located in the endoplasmic reticulum and Golgi complex (Figures 5C,D).

**Figure 6:**
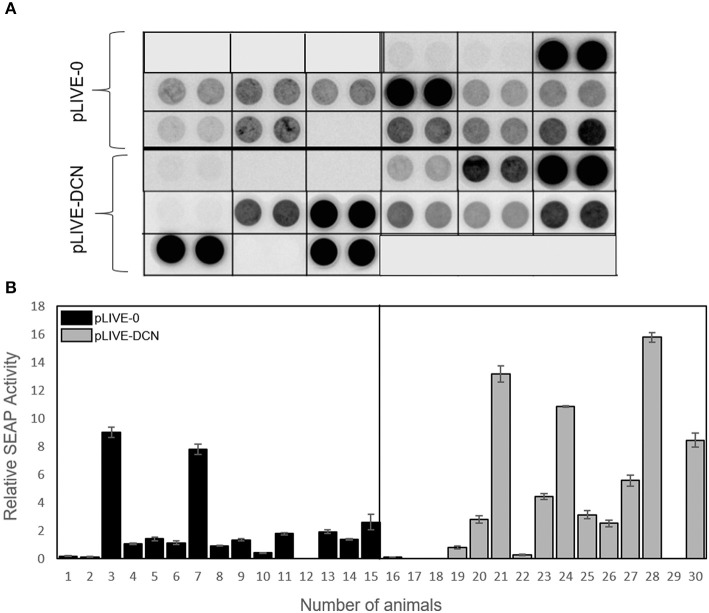
Measurement of secreted alkaline phosphatase activity from sera, after the injection with pLIVE-DCN and pLIVE-0 vectors. Serum was collected at half time of the TA treatment. SEAP chemiluminescent analysis **(A)** and its quantification **(B)**. The serum SEAP activity is indicated on the *y*-axis and the mice are indicated on the *x*-axis. *n* = 30. All data are demonstrated as mean of normalized ± SD.

As previously described, mice were injected with a plasmid encoding SEAP reporter gene. In most of the animals, the SEAP expression was high, measured from half-time TA treated mice blood samples (Figure 6). Very low SEAP level was detected in three of control (pLIVE-0), and four of decorin treated (pLIVE-DCN) animals.

**Figure 7:**
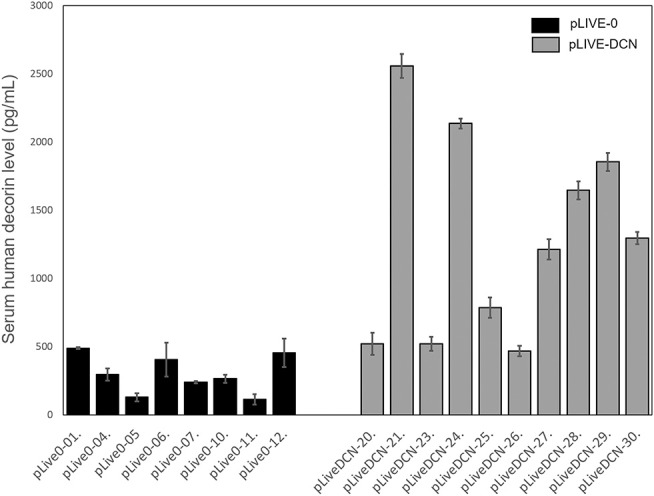
Decorin expression in control (pLIVE-0) and decorin treated (pLIVE-DCN) groups. The decorin expression is indicated on the *y*-axis (pg/ml) and the mice are indicated on the *x*-axis (*n* = 20). Data are indicated as mean of normalized ± SD.

Human recombinant decorin level was measured from the sera of mice by ELISA. The results correlated well with that of SEAP assay indicating that decorin delivery was successful and the proteoglycan production is active (Figure 7).

### Decorin Gene Transfer Effectively Diminishes Liver Carcinogenesis in Mice

**Figure 8:**
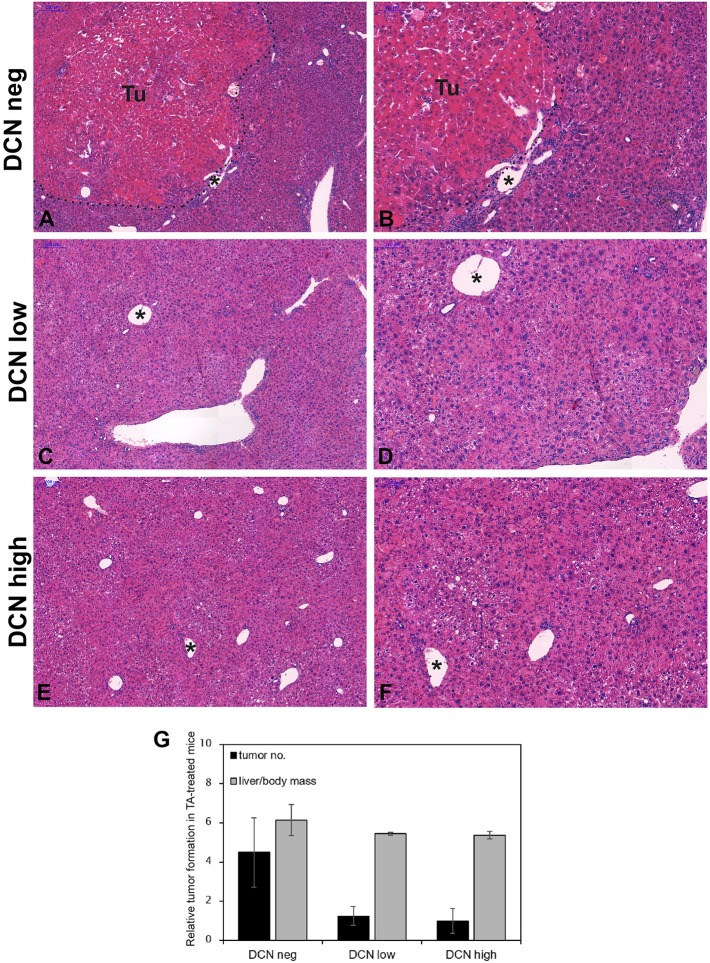
Representative histological images of hematoxylin and eosin-stained normal **(A,B)**, low **(C,D)**, and high **(E,F)** decorin expressing liver tissues induced by TA treatment. Tu, tumor; pointed lines indicate tumor border. Asterisks show the same vein in different magnifications. **(A,C,E)** Scale bar = 200 μm, **(B,D,F)** 100 μm. Bar charts represent the ratios of tumor-bearing mice in experimental groups of normal, low, and high decorin expressing groups with TA treatment **(G)**. *n* = 15. All data are indicated as mean of normalized ± SD.

Depending on the transfection efficiency measured by SEAP assay, decorin transfected group was subdivided into decorin negative (Figures 8A,B), low (Figures 8C,D), and high (Figures 8E,F) decorin expressing categories. Similarly to our previous studies (42) TA-induced fibrosis and subsequently hepatic cirrhosis led to hepatocellular cancer. Large tumorous nodules with abundant cytoplasm and strong eosinophilic staining surrounded by a connective tissue capsule were detected in control livers (Figures 8A,B). Upon TA-induced hepatocarcinogenesis, decorin transfection resulted in attenuated tumor formation in both low and high decorin expressing groups (Figure 8G). The highest tumor count was observed in mice with no decorin production (Figures 8A,B,G). Decorin delivery decreased the number of tumors by 72 and 78% in low and high decorin expressing groups respectively (Figure 8G) compared to decorin negative livers. Lower liver mass/body mass ratios of decorin treated animals corroborates the beneficial effect of the excessive proteoglycan. Based on these results, we assume that decorin gene delivery has the potential to inhibit the development of HCC indicating that soluble decorin may act as a tumor suppressor.

### Major Signaling Pathways Mediated by Overexpressed Decorin in HCC

**Figure 9:**
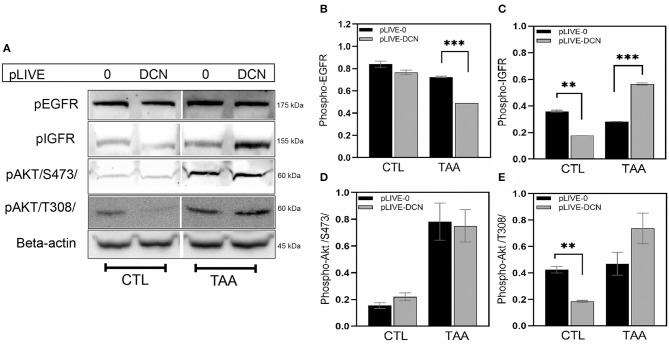
Representative images of Western blot membranes **(A)**. Bar charts show the relative levels of pEGFR **(B)**, pIGFR **(C)**, and Akt phosphorylation at Ser473 **(D)** and Thr308 **(E)** in lysates of wild-type (pLIVE-0) and decorin overexpressed (pLIVE-DCN) livers after TA exposure and without treatment (CTL, control). β-actin was used as loading control. All data are presented as mean of normalized ± SD. **p < 0.01; ***p < 0.001.

As several publications reflected that soluble decorin acts as a pan-RTK inhibitor targeting a multitude of RTKs, their activity in our experimental hepatocarcinogenesis model was tested. Among others, we detected decreased level of phospho-EGFR in pLIVE-DCN samples by 32% in TA treated groups relative to that of pLIVE-0 (*p* < 0.001, Figures 9A,B). IGF-IR activity did not alter upon TA exposure in pLIVE-0 animals (Figures 9A,C). However, we found a 2-fold increase in tyrosine phosphorylation of IGF-1R in DCN overexpressing TA-driven tumors (*p* < 0.001, Figures 9A,C).

As Akt is a known downstream effector of IGF-1R, we tested whether the levels of phospho-Akt (S473) and phospho-Akt (T308) would be altered in our experimental animal model. In control lysates, hardly any phospho-Akt (S473) was detected (Figures 9A,D), but phospho-Akt (T308) was ~2.3-fold higher in pLIVE-0 mice than in pLIVE-DCN samples (*p* < 0.01, Figures 9A,D,E). Upon TA exposure, their amount raised, and no difference was observed in pAkt (S473) level between the transfected groups (Figures 9A,D). In contrast, pAkt (T308) exhibited ~1.5-fold increase in pLIVE-DCN mice compared to the pLIVE-0 group (Figures 9A,D).

**Figure 10:**
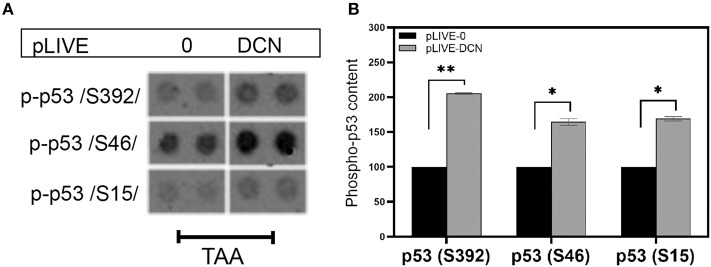
**(A)** Changes in phospho-p53/S392/, phospho-p53/S46/, and phospho-p53/S15/ in TA-induced liver cancer. **(B)** Representative image of the phospho-kinase array dots of different phospho-p53 forms in livers of wild-type (pLIVE-0) and decorin overexpressing (pLIVE-DCN) animals after TA exposure. Column represents the results of densitometry of array dots showing relative levels. All data are demonstrated as mean of normalized ± SD. *p < 0.05; **p < 0.01.

In our experimental hepatocarcinogenesis model changes in p53 levels were identified by a phospho-array study. Three phosphorylated p53, namely phospho-p53(S392), phospho-p53(S46), and phospho-p53(S15) exhibited significantly higher levels in response to the overexpressed of decorin (Figure 10). Notably, after TA exposure, we found ~2-fold, ~1.6-fold, and ~1.7-fold increase in phospho-p53(S392), phospho-p53(S46), and phospho-p53(S15) in decorin transfected mice compared to that of null-vector, respectively (*p* < 0.05 and 0.01, Figure 10).

## Discussion

Hepatocarcinogenesis is a multi-step process characterized by progressive cellular and molecular changes of hepatocytes and leads to the emergence of HCC (45). Studies of the last decade revealed that the tumor microenvironment is an active participant of the tumor development and adds another factor that should be considered in the study of its pathology. The main non-cellular component of these events is the ECM build up by various macromolecules (proteins, glycoproteins, proteoglycans, and glycosaminoglycans) with different biochemical properties and biological functions (46).

Decorin, a SLRP expressed by fibroblast and myofibroblasts, is an integrated member of ECM (47). Multitude reports on human cancers provided evidences about the tumor suppressor potential of this proteoglycan. Its action is related to the inhibition of RTKs (20, 48, 49). Regarding liver tumors, there is hardly any data on the role of decorin in the literature (14, 15, 42, 50–54). To fill up this hiatus we hypothesized that decorin may act as a tumor suppressor in HCC. To challenge our presumption a four-step model system was designed: (1) we examined the mRNA expression of decorin in HCC using *in silico* approaches; (2) FFPE TMA samples of HCC with or without cirrhosis were applied to measure decorin content at protein level; (3) cell culture experiments were to test whether tumor cells can inhibit decorin production of myofibroblasts; and (4) animal experiments were designed to clarify the potential of decorin to inhibit the development of HCC evoked by TA.

In previous studies, only absolute decorin expression has been measured in a variety of tumors. Here, we normalized decorin expression to SMA content in order to compensate for the variation of fibroblast content, α-SMA is a well-known marker for activated myofibroblasts (55), which are the key producer of decorin. We performed gene expression profiling of liver specimens with or without cancer using a Dataset E-MTAB-950 [containing 34 normal, 112 tumors, and 5 pairs of tumor–non-tumorous adjacent tissue (NAT)] from ArrayExpress database. Decorin expression was significantly downregulated in most HCCs compared to their NATs and to the normal liver. In addition, mRNA expression analysis revealed that normalized DCN content seems to follow the staging of HCC. Indeed, transcriptional analysis of tumor progression at the mRNA level revealed high decorin expression during the early stages of tumorigenesis in B-cell chronic lymphoid leukemia (CLL), in contrast to its suppression in advanced stages (56). Similarly, while benign hemangiomas displayed relatively high decorin mRNA levels, the transcription of decorin was completely blocked in malignant vascular sarcomas (57). Therefore, it seems that malignant behavior and tumor progression may be correlated with the loss of endogenous decorin expression. Our study indicates that reduced expression of decorin is associated with decorin gene downregulation and is irrespective of the number of myofibroblasts.

Next, TMA was assembled from HCC FFPE samples to determine their decorin content. Microarray slides were immunostained for decorin and α-SMA, and decorin level was normalized to SMA content, as seen in *in silico* approaches. In general, HCC tumor tissues have reduced or completely blocked decorin expression compared to their paired peritumoral liver areas. In contrast to the *in silico* results, neither the relative amount of decorin mRNA, nor its protein level was downregulated in tumor samples compared to normal tissue. Our different control samples may be responsible for this result. In our TMA assembly, we used hemangiomas as a control group, while there is no information about the control of *in silico* database. They could be healthy livers, or other non-tumorous tissue, which may affect the expression of decorin.

An important issue is that decorin mainly exists in collagen-bound form. However, only its soluble variant can bind and inhibit tyrosine kinases receptors, such as EGFR, Met, IGF-1R, VEGFR, and PDGFR (14, 37, 39, 40, 47, 58). Interestingly, there is no significant difference between HCCs with or without cirrhosis, decorin expression is reduced or completely blocked in both types of tumors compared to NAT. In our sample set 52% of HCCs were decorin negative, 33% showed low, and 15% high decorin expression. Negativity and low expression were more characteristic for HCCs without cirrhosis. These results suggest that the lack of the proteoglycan provides a survival advantage for the tumor tissue. The mechanism of this downregulation needs further studies to elucidate. In tumor tissues, the decreased expression of decorin may related to methylated DCN gene. Qian et al. (59) have identified the methylated +58CpG in DCN 5′-UTR associated with reduced expression of DCN mRNA in non-small cell lung cancer. According to the Human Protein Atlas database, decorin level is significantly reduced in various tumor types compared to their non-tumorous tissue pair (60). They found that decorin is strongly expressed in the peritumoral stroma, and the proteoglycan level is markedly diminished or disappeared in the tumor stroma (60). Decreased decorin expression was observed in urothelial carcinoma, skin squamous and basal cell carcinoma, mammary lobular and ductal carcinoma, cervix adenocarcinoma, serous or mucinous cystadenocarcinoma and ovarium endometrioid carcinoma, colon, kidney, pancreas, prostate, rectum and the stomach, and embryonal carcinoma and seminoma of the testis (60). It was also shown that decorin gene is under-expressed by at least 50% in lung adenocarcinomas and squamous cell carcinomas compared to normal tissue (61). To test whether tumor cells are capable of directly influencing the decorin production of myofibroblasts, LX2 human stellate cells were exposed to hepatoma conditioned media. Significantly, less decorin was detected in the media of LX2 cells when HLE, HepG2, or Huh7 conditioned media was applied (Figure 4). These results corroborate our observations in human HCC tissue samples. Similar results were obtained by Van Bockstal et al. (62), where decorin expression of cancer associated fibroblasts was significantly reduced when conditioned media of breast cancer cell lines were applied. In their experiments, TGFβ1 was identified as the main inducer of decorin repression in cancer associated fibroblasts. Indeed, TGFβ1 is a known transcriptional inhibitor of DCN gene (63), and the hepatoma cell lines applied produce considerable amounts of the cytokine (53). Thus, it is conceivable that the production of TGFβ1 by tumor cells is responsible for decorin downregulation in the neighboring fibroblasts. The fact that the presence of tumor cells reduces the expression of decorin highlights its tumor suppressor effect in HCC and further studies are needed to unravel the exact silencing mechanism of this SLRP.

Our previous studies showed that the lack of decorin favors primary hepatocarcinogenesis, which results in higher tumor incidence (42), however it was a further question whether the addition of the proteoglycan is able to counteract primary hepatocarcinogenesis evoked by TA. Thus, we planned a new set of investigations, where targeted delivery of decorin to the liver has been carried out. The excessive proteoglycan was expressed by hepatocytes, mainly around central veins (Figure 5). Upon thioacetamide exposure the highest tumor number was observed in animals with no excessive decorin production (Figure 8). Our findings are in line with a vast number of earlier studies, where delivery of decorin via an adenovirus vector into the tumor cells inhibited the growth of lung, colon, and squamous cell carcinomas by attenuating EGFR phosphorylation (64). In addition, decorin transfer inhibited Met and Wnt/β-catenin signaling pathways and thus prevented the formation of bone metastasis of prostate cancer cells (65). Virus-delivered decorin attenuated breast cancer growth and prevented its metastasis formation in various organs (66–68). Ma et al. (69) found that decorin gene therapy prolonged survival and inhibited tumor growth in an *in vivo* glioma model. The rate of inhibition directly correlated with the expression levels of decorin and with the timing of DCN gene transfer. Decorin gene therapy was successfully applied in models of prostate and pancreatic cancers as well (70, 71). Our results together with these studies confirm that elevated decorin expression *in vivo* is able to protect against tumorigenesis, as well as other way around, its downregulation in tumorous stroma stimulates tumor invasion. The protective role of decorin in experimental situations raises the possibility that this proteoglycan can be utilize in the battle against human cancer.

As an unexpected result, in contrast with other tyrosine kinases receptors we observed striking activation of IGF-1R followed by Akt phosphorylation upon TA exposure in pLIVE-DCN groups. In parallel, elevated phospho-p53 levels were observed especially of that phosphorylated at serine 46 residue a marker of cell death response (72). This finding is in agreement with the publication shown that decorin can activate this receptor inducing Akt phosphorylation in renal fibroblasts and normal endothelial cells (38). Similarly, in our earlier experiments we found that addition of human recombinant decorin to the Hep3B hepatoma cell line provoked activation of IGF-1R and insulin receptors and massive Akt activation (53). An earlier study reported that IGF-1R exerts a supportive function in apoptosis mediated by p53 (73). In addition, adenovirus-delivered decorin expression was proved to provoke cell death via activation of p53 (74). Thus, it is conceivable that excessive decorin curbs tumorigenesis via induction of IGF-1R that in turn induces apoptosis via p53.

In conclusion, our results suggest that decorin plays a protective role in liver cancer. Theoretically, utilization of decorin as a physiological tyrosine kinase receptor inhibitor, targeting multiple receptors, is possible and the idea is well-established.

## Data Availability Statement

Publicly available datasets were analyzed in this study. This data can be found here: Gene expression datasets: ArrayExpress database, provided by the European Bioinformatics Institute (Saffron Walden, UK). Our datasets with accession E-MTAB-950 includes 36 normal, 112 tumors, and 10 pair of tumors–non-tumorous adjacent tissues.

## Ethics Statement

The studies involving human participants were reviewed and approved by Semmelweis University Regional and Institutional Committee of Science and Research Ethics (TUKEB permit number: 95/1999). The patients/participants provided their written informed consent to participate in this study. The animal study was reviewed and approved by Animal Health Care and Control Institute, Csongrád County, Hungary All of the experiments were approved by the following ethical license: XVI/ 03047-2/2008.

## Author Contributions

AR, ZH, and HF conducted the experiments and drafted the manuscript. BW performed *in silico* experiments, statistical analyses, and revised the manuscript. PT have performed language revision and made intellectual contribution to the work. IK and KB designed the research and revised the manuscript. All the authors have read the final manuscript and approved its publication.

## Conflict of Interest

PT was employed by the company Solvo Biotechnology. The remaining authors declare that the research was conducted in the absence of any commercial or financial relationships that could be construed as a potential conflict of interest.

## Abbreviations

BCLC: Barcelona Clinic Liver Cancer staging
BSA: bovine serum albumin
cDNA: complementary deoxyribonucleic acid
CM: conditioned medium
DAB: 3,3-diaminobenzidine tetrahydrochloride
DAPI: 4′,6-diamidino-2-phenylindole
DCN: decorin
DMEM: Dulbecco's modified Eagle's medium
ECM: extracellular matrix
EDTA: ethylenediaminetetraacetic acid
EGFR: epidermal growth factor receptor
ELISA: enzyme-linked immunosorbent assay
ErbB2: receptor tyrosine-protein kinase erbB-2; erythroblastic oncogene B
FBS: fetal bovine serum
FFPE: formalin fixed paraffin embedded tissue
GAPDH: glyceraldehyde 3-phosphate dehydrogenase
HBV: hepatitis B virus
HCC: hepatocellular carcinoma
HCV: hepatitis C virus
HSCs: hepatic stellate cells
IGF-IR: insulin-like growth factor 1 receptor
MFs: myofibroblasts
mRNA: messenger ribonucleic acid
NAT: non-tumorous adjacent tissues
PBS: phosphate buffered saline
PDGFR: platelet-derived growth factor receptor
PG: proteoglycan
pLIVE: Liver *in vivo* Expression vector
PVDF: polyvinylidene difluoride
RTK: receptor tyrosine kinase
RT-qPCR: Reverse Transcription PCR
SEAP: secreted alkaline phosphatase
SLRP: small leucine rich proteoglycan
SMA: smooth muscle actin
TA: thioacetamide
TBS: tris buffered saline
TGF-β: transforming growth factor beta
TMA: tissue microarray
VEGFR: vascular endothelial growth factor receptor.
