# Supplementary material for: Protective Role of Decorin in Primary Hepatocellular Carcinoma
Source: Front Oncol. 2020 May 12;10:645. doi: 10.3389/fonc.2020.00645 (PMC7235294; doi:10.3389/fonc.2020.00645)
Supplement: Supplementary file 1 [file Table_1.docx]

**Supplementary material**

**Table S1. List of biopsy samples**

| **HCC samples with chirrosis** | | | | |
| --- | --- | --- | --- | --- |
| **Patient ID** | **Sex** | **Age** | **Tissue type** | **Etiology** |
| 01 | male | 1964 | with cirrhotic | HCV |
| 02 | female | 1953 | with cirrhotic | HCV |
| 03 | male | 1947 | with cirrhotic | HCV |
| 04 | male | 1940 | with cirrhotic | unknown |
| 05 | male | 1941 | with cirrhotic | unknown |
| 06 | female | 1957 | with cirrhotic | unknown |
| 07 | female | 1935 | with cirrhotic | HCV |
| 08 | male | 1960 | with cirrhotic | HCV |
| 09 | male | 1954 | with cirrhotic | HCV |
| 10 | male | 1963 | with cirrhotic | HCV |
| 11 | female | 1962 | with cirrhotic | HCV |
| 12 | female | 1958 | with cirrhotic | NASH |
| 13 | male | 1952 | with cirrhotic | aethyl |
| 14 | male | 1944 | with cirrhotic | HCV |
| 15 | male | 1944 | with cirrhotic | HBV |
| 16 | female | 1937 | with cirrhotic | HCV |
| 17 | male | 1985 | with cirrhotic | hepatoblastoma |
| 18 | male |  | with cirrhotic | HBV, aethyl |
| 19 | male |  | with cirrhotic | HCV |
| **HCC samples without chirrosis** | | | | |
| 20 | male | 1943 | non-cirrhotic | unknown |
| 21 | female | 1942 | non-cirrhotic | unknown |
| 22 | male | 1945 | non-cirrhotic | unknown |
| 23 | male | 1982 | non-cirrhotic | unknown |
| 24 | male | 1988 | non-cirrhotic | adenoma |
| 25 | male | 1926 | non-cirrhotic | unknown |
| 26 | male | 1944 | non-cirrhotic | unknown |
| 27 | male | 1961 | non-cirrhotic | HCV |
| 28 | male | 1933 | non-cirrhotic | unknown |
| **Control samples** | | | | |
| 29 | female | 1973 | haemangioma |  |
| 30 | female | 1969 | haemangioma |  |
| 31 | female | 1972 | haemangioma |  |
| 32 | female | 1969 | haemangioma |  |
| 33 | female | 1959 | haemangioma |  |
| 34 | female | 1947 | haemangioma |  |
| 35 | female | 1978 | haemangioma |  |
| 36 | female | 1972 | haemangioma |  |
| 37 | female | 1975 | haemangioma |  |

**Table S2.** **Antibodies used in the present study.**

| **Antigen specificity** | **Species** | **Manufacturer*** | **Cat.No** | **Dilution for IHC** | **Dilution for WB/DB** |
| --- | --- | --- | --- | --- | --- |
| Decorin | Rabbit Polyclonal | Sigma-Aldrich | HPA003315 | 1:50 | - |
| Smooth Muscle Actin (SMA) | Mouse Monoclonal | DakoCytomation | m0851 | 1:50 | - |
| EGFR | Rabbit Polyclonal | Sigma-Aldrich | HPA018530 | - | 1:1000 |
| Phospho-EGFR (Tyr1068) | Rabbit monoclonal | Cell Signaling | 3777 | - | 1:1000 |
| Phospho-IG1FR (Y1161) | Rabbit Polyclonal | Abcam | ab39398 | - | 1:1000 |
| Phospho-Akt (Thr308) | Rabbit monoclonal | Cell Signaling | 2965 | - | 1:1000 |
| Phospho-Akt (S473) | Rabbit monoclonal | Cell Signaling | 4058 | - | 1:1000 |
| Β-actin | Mouse monoclonal | Sigma-Aldrich | A2228 | - | 1:5000 |
| **Secondary Antibodies** | **Species** | **Manufacturer*** | **Cat.No** | **Dilution for IHC** | **Dilution for WB/DB** |
| Anti-Rabbit immunglobulin/HRP | Goat Polyclonal | DakoCytomation | P 0448 | 1:200 | 1:2000 |
| Anti-Mouse Immunglobulin/HRP | Goat Polyclonal | DakoCytomation | P 0447 | 1:200 | 1:2000 |
| Alexa Fluor® 568, anti-Rabbit IgG | Goat Polyclonal | Invitrogen | a-11011 | 1:200 | - |

* DakoCytomation Glostrup Denmark, Invitrogen/Life Technologies Carlsbad CA, Sigma-Aldrich St. Louis, MO, Abcam Cambrige UK, Cell Signaling Technology Danvers, MA

**Table S3. Results of decorin immunostainings on HCC TMA.**

| **Patient ID** | **Tissue** | **Decorin score** | **SMA score** | **DCN/SMA score** | **Category** |
| --- | --- | --- | --- | --- | --- |

| **HCC samples with chirrosis** |
| --- |

| 01 | Tumor | 1.5 | 1 | 1.5 | DCN low |
| --- | --- | --- | --- | --- | --- |
| 01 | NAT | 8 | 6 | 1.3 |  |
| 02 | Tumor | 0 | 2 | 0,0 | DCN negative |
| 02 | NAT | 11 | 8 | 1.4 |  |
| 03 | Tumor | 0 | 4 | 0,0 | DCN negative |
| 03 | NAT | 11 | 9.5 | 1.2 |  |
| 04 | Tumor | 0 | 9 | 0,0 | DCN negative |
| 04 | NAT | 9.5 | 7.5 | 1.3 |  |
| 05 | Tumor | 0 | 4 | 0.0 | DCN negative |
| 05 | NAT | 11.5 | 10 | 1.2 |  |
| 06 | Tumor | 1.5 | 8.5 | 0.2 | DCN low |
| 06 | NAT | 12 | 11.5 | 1.0 |  |
| 07 | Tumor | 0 | 4 | 0.0 | DCN negative |
| 07 | NAT | 10.5 | 10 | 1.1 |  |
| 08 | Tumor | 2 | 1.5 | 1.3 | DCN low |
| 08 | NAT | 12 | 12 | 1.0 |  |
| 09 | Tumor | 1 | 4,5 | 0,2 | DCN low |
| 09 | NAT | 11.5 | 7 | 1.6 |  |
| 10 | Tumor | 5 | 11.5 | 0.4 | DCN low |
| 10 | NAT | 11.5 | 12 | 1.0 |  |
| 11 | Tumor | 1.5 | 4.5 | 0.3 | DCN low |
| 11 | NAT | 3.5 | 7.5 | 0.5 |  |
| 12 | Tumor | 11.5 | 8.5 | 1.4 | DCN high |
| 12 | NAT | 11.5 | 9 | 1.3 |  |
| 13 | Tumor | 0 | 5.5 | 0.0 | DCN negative |
| 13 | NAT | 12 | 8 | 1.5 |  |
| 14 | Tumor | 3.5 | 9.5 | 0.4 | DCN low |
| 14 | NAT | 11.5 | 9 | 1.3 |  |
| 15 | Tumor | 0 | 4.5 | 0.0 | DCN negative |
| 15 | NAT | 3 | 6 | 0.5 |  |
| 16 | Tumor | 11.5 | 12 | 1.0 | DCN high |
| 16 | NAT | 11 | 8 | 1.4 |  |
| 17 | Tumor | 0 | 7 | 0.0 | DCN negative |
| 17 | NAT | 11.5 | 8 | 1.4 |  |
| 18 | Tumor | 7 | 12 | 0,6 | DCN high |
| 18 | NAT | 12 | 12 | 1.0 |  |
| 19 | Tumor | 0 | 4 | 0.0 | DCN negative |
| 19 | NAT | 12 | 5 | 2.4 |  |

| **HCC samples without chirrosis** |
| --- |

| 20 | Tumor | 5 | 12 | 0.4 | DCN low |
| --- | --- | --- | --- | --- | --- |
| 20 | NAT | 1 | 1.5 | 0.7 |  |
| 21 | Tumor | 0 | 4 | 0.0 | DCN negative |
| 21 | NAT | 11 | 7 | 1.6 |  |
| 22 | Tumor | 0 | 11.5 | 0.0 | DCN negative |
| 22 | NAT | 1 | 1.5 | 0.7 |  |
| 23 | Tumor | 7 | 9.5 | 0.7 | DCN high |
| 23 | NAT | 7 | 12 | 0.6 |  |
| 24 | Tumor | 0 | 5 | 0.0 | DCN negative |
| 24 | NAT | 5.5 | 12 | 0.5 |  |
| 25 | Tumor | 0 | 9 | 0.0 | DCN negative |
| 25 | NAT | 6.5 | 7.5 | 0.9 |  |
| 26 | Tumor | 1 | 9.5 | 0.1 | DCN low |
| 26 | NAT | 9 | 8.5 | 1.1 |  |
| 27 | Tumor | 0 | 4 | 0.0 | DCN negative |
| 27 | NAT | 12 | 7.5 | 1.6 |  |
| 28 | Tumor | 0 | 4.5 | 0.0 | DCN negative |
| 28 | NAT | 2 | 4.5 | 0.4 |  |
| **Control samples** | | | | | |
| 29 | Control | 0.5 | 7.5 | 0.1 |  |
| 30 | Control | 0.5 | 1.5 | 0.3 |  |
| 31 | Control | 0.5 | 2.5 | 0.2 |  |
| 32 | Control | 1 | 3.5 | 0.3 |  |
| 33 | Control | 1 | 6.5 | 0.2 |  |
| 34 | Control | 2.5 | 6.5 | 0.4 |  |
| 35 | Control | 1 | 3.5 | 0.3 |  |
| 36 | Control | 0.5 | 1 | 0.5 |  |
| 37 | Control | 1 | 2.5 | 0.4 |  |
